# Argonaute 2 is lost from neuromuscular junctions affected with amyotrophic lateral sclerosis in SOD1^G93A^ mice

**DOI:** 10.1038/s41598-022-08455-y

**Published:** 2022-03-17

**Authors:** Dillon Shapiro, Ryan Massopust, Thomas Taetzsch, Gregorio Valdez

**Affiliations:** 1 Molecular Biology, Cell Biology, & Biochemistry Graduate Program, Brown University, Providence, RI USA; 2 Department of Molecular Biology, Cell Biology and Biochemistry, Brown University, 70 Ship St, Providence, RI 02903 USA; 3 Center for Translational Neuroscience, Robert J. and Nancy D. Carney Institute for Brain Science and Brown Institute for Translational Science, Brown University, Providence, RI USA; 4 Department of Neurology, Warren Alpert Medical School of Brown University, Providence, USA

**Keywords:** Amyotrophic lateral sclerosis, Neuromuscular junction, Molecular neuroscience, miRNAs

## Abstract

miRNAs are necessary for neuromuscular junction (NMJ) health; however, little is known about the proteins required for their activity in this regard. We examined expression of Argonaute 2 (Ago2) and miRNA biogenesis genes in skeletal muscles during development, following nerve injury and in the SOD1^G93A^ ALS mouse model. We found that these genes are enriched in neonate muscles and in adult muscles following nerve injury. Despite widespread NMJ deterioration, these genes were not increased in muscles of SOD1^G93A^ mice. We also found that Ago2 distribution is linked to maturation, innervation, and health of NMJs. Ago2 increasingly concentrates in synaptic regions during NMJ maturation, disperses following experimental denervation and reconcentrates at the NMJ upon reinnervation. Similar to experimentally denervated muscles, a homogenous distribution of Ago2 was observed in SOD1^G93A^ muscle fibers. To determine if Ago2 is necessary for the health of adult muscles, we excised Ago2 from Ago2^fl/fl^ mice using adeno-associated virus mediated Cre recombinase expression. We observed modest changes in muscle histology after 3 months of Ago2 knockdown. Together, these data provide critical insights into the role of Ago2 and miRNA biogenesis genes in healthy and ALS-afflicted skeletal muscles and NMJs.

## Introduction

MicroRNAs (miRNAs) are 20–25 nucleotide RNAs that are ubiquitously expressed in mammalian cells where they mainly mediate post-transcriptional gene regulation in the cytosol^1^. In doing so, miRNAs provide rapid, localized gene regulation that is particularly important for large cells with specialized regional functions, such as skeletal muscle fibers. miRNAs rely on a number of RNA binding proteins to carry out post-transcriptional gene regulation. These include the miRNA biogenesis proteins Drosha, DGCR8, exportin-5 and Dicer, which are necessary for processing and transporting nascent primary miRNAs in the nucleus into mature single-stranded miRNAs that intercept target transcripts in the cytosol^1–3^. Sequestration of target mRNAs requires the interaction of a single miRNA with the RNA binding protein Argonaute (Ago) in what is known as the RNA induced silencing complex (RISC). Base pair interactions between a miRNA and the 3′ UTR of the target mRNA guide Ago to transcripts that are destined to be silenced^1,2^. Ago then catalyzes RISC activity by binding and either sequestering or degrading target mRNAs^1,2^. Four Ago homologues (Ago1-4) exist in mammals, among which Ago2 is the prominent mediator of RISC activity and the only homologue with endonuclease capability^1^. Underscoring its prominence in mediating post-transcriptional gene regulation, Ago2 deletion in mice is embryonic lethal^4^. Moreover, Ago2 has been shown to mediate neurological development^4,5^, synaptic plasticity^6,7^ and muscle regeneration^8^.

In recent decades a growing number of muscle-specific miRNAs have been demonstrated to be instrumental to the development and health of muscle fibers and their NMJs^9,10^. A subset of these miRNAs have been shown to be differentially regulated in skeletal muscles of amyotrophic lateral sclerosis (ALS) patients^11^ and to influence neuromuscular junction (NMJ) health in mice harboring ALS-causing mutant genes^12–14^. For example, miR-206 is upregulated by muscles in symptomatic mice harboring the ALS-causing G93A mutation in the superoxide dismutase 1 gene (SOD1^G93A^)^14,15^, while deletion of miR-206 accelerates NMJ degeneration and reduces survival in these mice^14^. Decreased levels of miR-126-5p in SOD1^G93A^ muscle have been linked to NMJ degeneration via loss of inhibition of the destabilizing factor semaphorin 3A^12^. Despite these advances in our understanding of the roles of miRNAs in skeletal muscle and NMJ health^9,10,13,14^, little is known about the proteins that are required for miRNA activity in skeletal muscles during development, injury, NMJ denervation or ALS.

In the current study, we examined the expression of Ago2 and miRNA biogenesis genes alongside the distribution of Ago2 in developing and adult skeletal muscles of healthy mice. We also assessed these genes following denervation in healthy adult mice and at different stages of ALS pathology in SOD1^G93A^ mice. We found that Ago2 and miRNA biogenesis genes are highly expressed in developing compared to adult skeletal muscles. During this period, Ago2 is widely distributed in both synaptic and non-synaptic regions of muscle fibers, however its distribution gradually concentrates at the NMJ by adulthood. In healthy young adult mice, we found that Ago2 expression is induced by experimental nerve injury, however synaptic concentration is temporally lost. By contrast, fast-twitch skeletal muscles collected from symptomatic SOD1^G93A^ mice, in which NMJ denervation is well-documented^16^, did not display elevated Ago2 expression but did display loss of synaptic Ago2 concentration. Supporting important roles for Ago2 in skeletal muscles, knockdown of Ago2 increased the presence of mononucleated cells in the interstitial space in young adult mice. These findings suggest that Ago2 plays important roles in the maintenance and repair of muscles and NMJs.

## Results

### Ago2 is enriched in developing skeletal muscle

**Figure 1:**
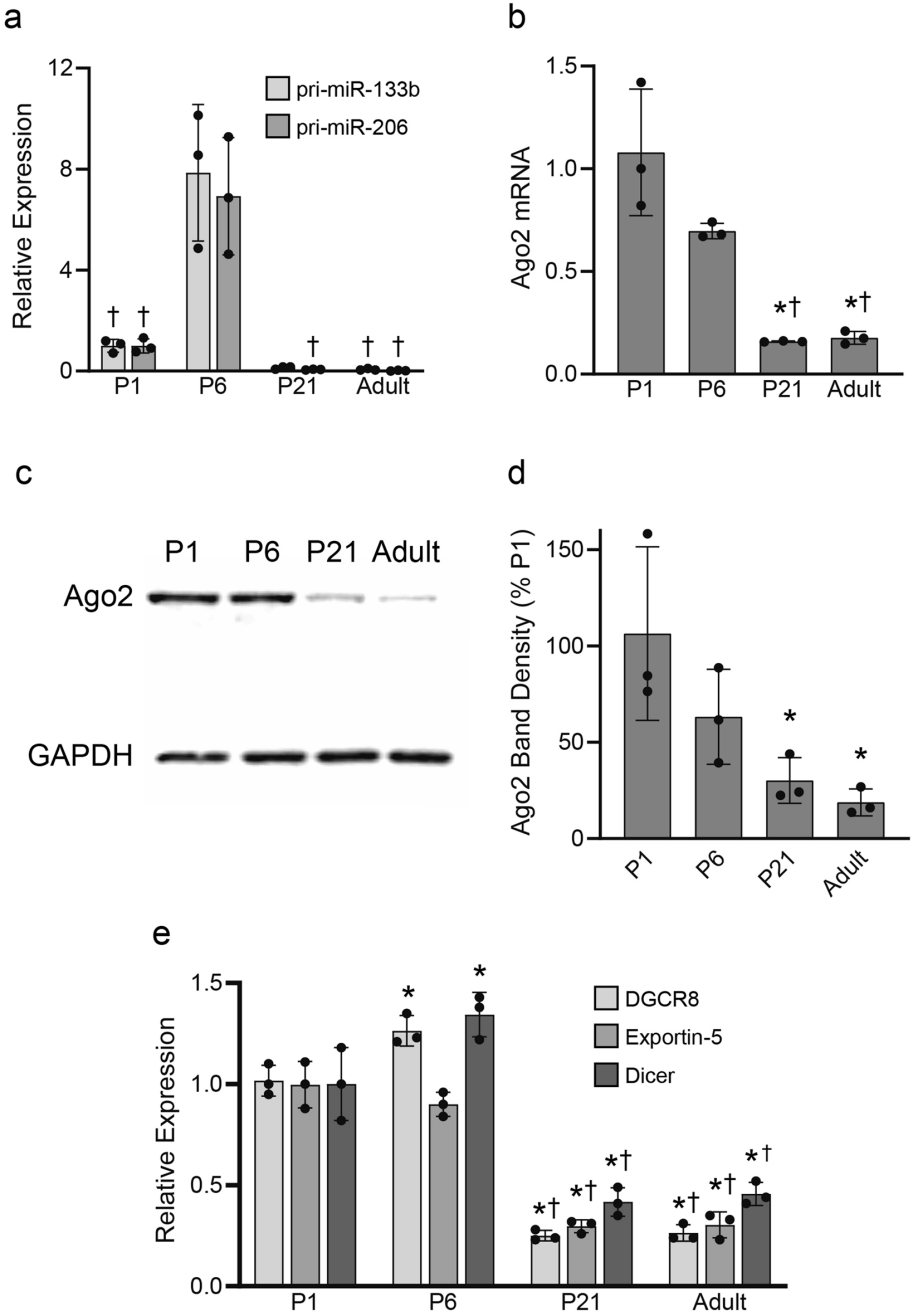
miRNA biogenesis gene expression is elevated in developing TA muscles. (**a**,**b**) qPCR analysis of pri-miRs 133b and 206 (**a**) and Ago2 mRNA (**b**) expression. (**c**,**d**) Western blot analysis of Ago2 protein levels. Images are cropped from the full-length blot, available in Fig. S5. (**e**) qPCR analysis of DGCR8, Exportin-5 and Dicer mRNA expression. *p < 0.05 versus P1. ^†^p < 0.05 versus P6. One-way ANOVA with Bonferroni post-hoc. All values reported as mean ± SD; n = 3.

Skeletal muscle-specific miRNAs are highly expressed during development where they regulate genes involved in myogenesis^9,10^. Confirming these previous findings, we found that levels of the pri-miR-133b and pri-miR-206 are elevated in the tibialis anterior (TA) and extensor digitorum longus (EDL) muscles of developing (P6) mice as compared to those from juvenile (P21) and adults aged 2–6 months (Fig. 1a). To determine whether Ago2 and miRNA biogenesis genes are similarly enriched during skeletal muscle development we performed gene expression analyses in the TA and EDL muscles collected from postnatal (P1 and P6), juvenile (P21) and adult (2–6 months old) wild type (WT) mice. We found that Ago2 expression is significantly upregulated in skeletal muscles at both the mRNA (Fig. 1b) and protein (Fig. 1c,d) levels in mice aged 1 and 6 days, as compared to juveniles and adults. Transcripts of the miRNA biogenesis genes DGCR8, Exportin-5 and Dicer were also highly upregulated in the TA and EDL muscles at the same developmental time points (Fig. 1e). These results demonstrate that Ago2 and miRNA biogenesis gene expression patterns mirror overall increases in miRNA expression during muscle development and maturation.

### Ago2 becomes concentrated at NMJs as they mature

**Figure 2:**
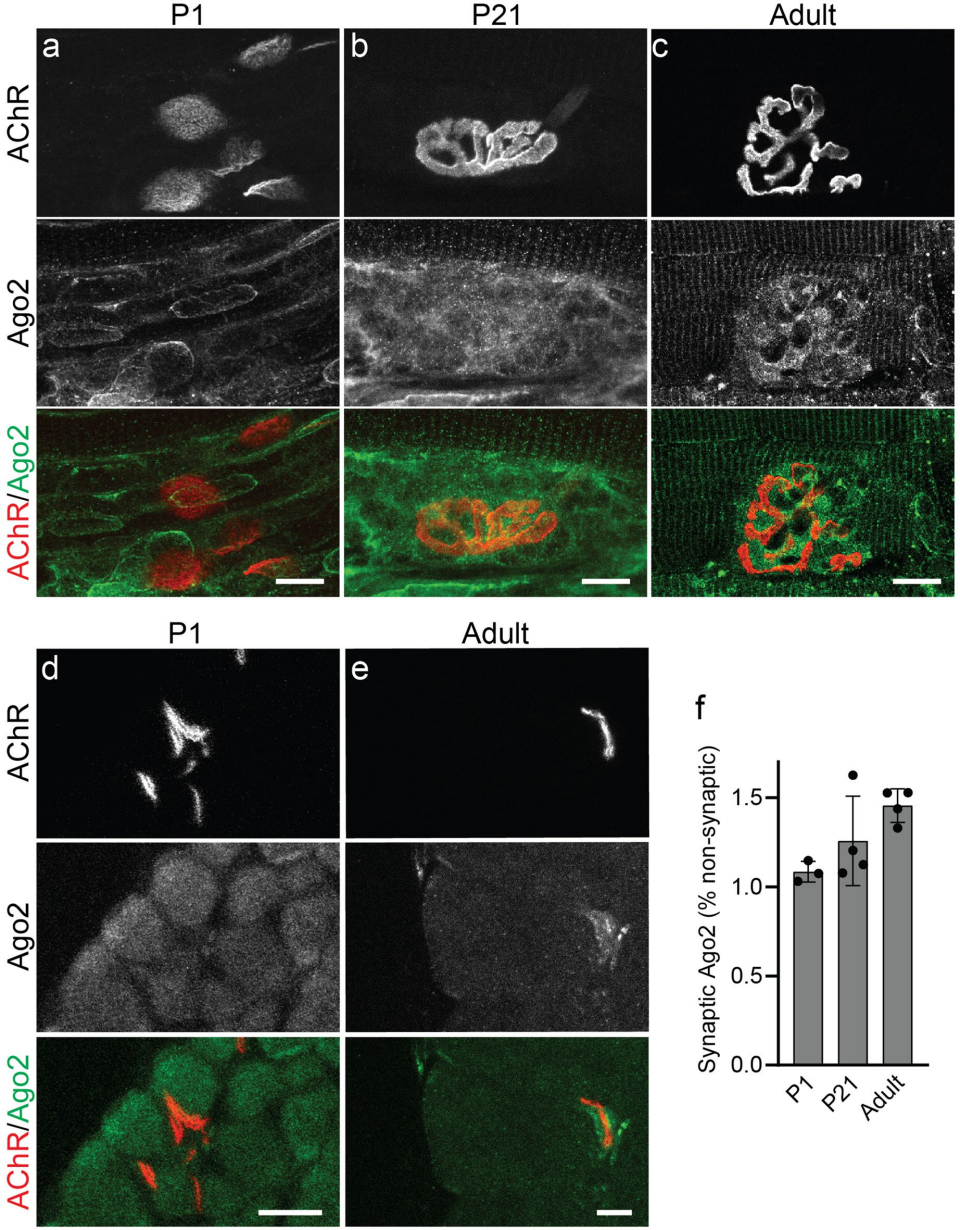
Ago2 distribution is concentrated at the NMJ in adult EDL muscle. (**a**–**c**) Representative images of Ago2 IHC (green) and fBTX labeled AChRs (red) in P1 (**a**), P21 (**b**) and Adult (**c**) EDL. (**d**,**e**) Representative images of Ago2 IHC (green) and fBTX labeled AChRs (red) in P1 (**d**) and Adult (**e**) TA cross-sections. (**f**) Quantification of synaptic Ago2 pixel intensity, relative to non-synaptic Ago2 pixel intensity, in TA muscle cross sections. *p < 0.05, unpaired 2-sided t test. All values reported as mean ± SD; n = 3–4. All scale bars = 10 µm.

Given that miRNAs participate in both skeletal muscle development^9,10^ and NMJ health^13,14,17,18^, we examined the distribution of Ago2 in synaptic and non-synaptic regions of muscle fibers in developing and mature skeletal muscles. To do so, we performed immunohistochemistry (IHC) for Ago2 on whole mount EDL muscles collected from P1, P21 and P60 mice. NMJs were identified by labeling nicotinic acetylcholine receptors (AChRs) with fluorescently conjugated α-Bungarotoxin (fBTX). We observed widespread distribution of Ago2 in both synaptic and non-synaptic regions of the developing EDL in P1 mice (Fig. 2a). By contrast, in juvenile and adult muscles Ago2 distribution was more pronounced in synaptic versus non-synaptic regions (Fig. 2b,c). To quantify our observed changes in Ago2 distribution, we performed Ago2 IHC on cross sections of the TA muscle (Fig. 2d,e) and made intra-fiber comparisons of Ago2 pixel intensity between synaptic regions and non-synaptic regions. During early postnatal development we observed an approximately 1:1 ratio of synaptic versus non-synaptic Ago2 pixel intensity (Fig. 2d,f). In adult muscles, however, a shift towards increased synaptic Ago2 pixel intensity was observed where the synaptic to non-synaptic ratio approached 1.5:1.0 (Fig. 2e,f). To determine whether increases in synaptic Ago2 in adult muscle are due to its localization at the NMJ pre- or postsynapse, we examined serial confocal optical sections of NMJs and constructed orthogonal projections to delineate the NMJ pre- and postsynapse. We found that Ago2 is predominately distributed along the NMJ postsynapse where it intercalates between AChR clusters (Fig. S1). These results suggest that Ago2 is diffusely distributed throughout muscles fibers during development and becomes increasingly concentrated near AChR clusters of the NMJ postsynapse as muscles mature.

### Skeletal muscles respond to denervation by modulating Ago2

**Figure 3:**
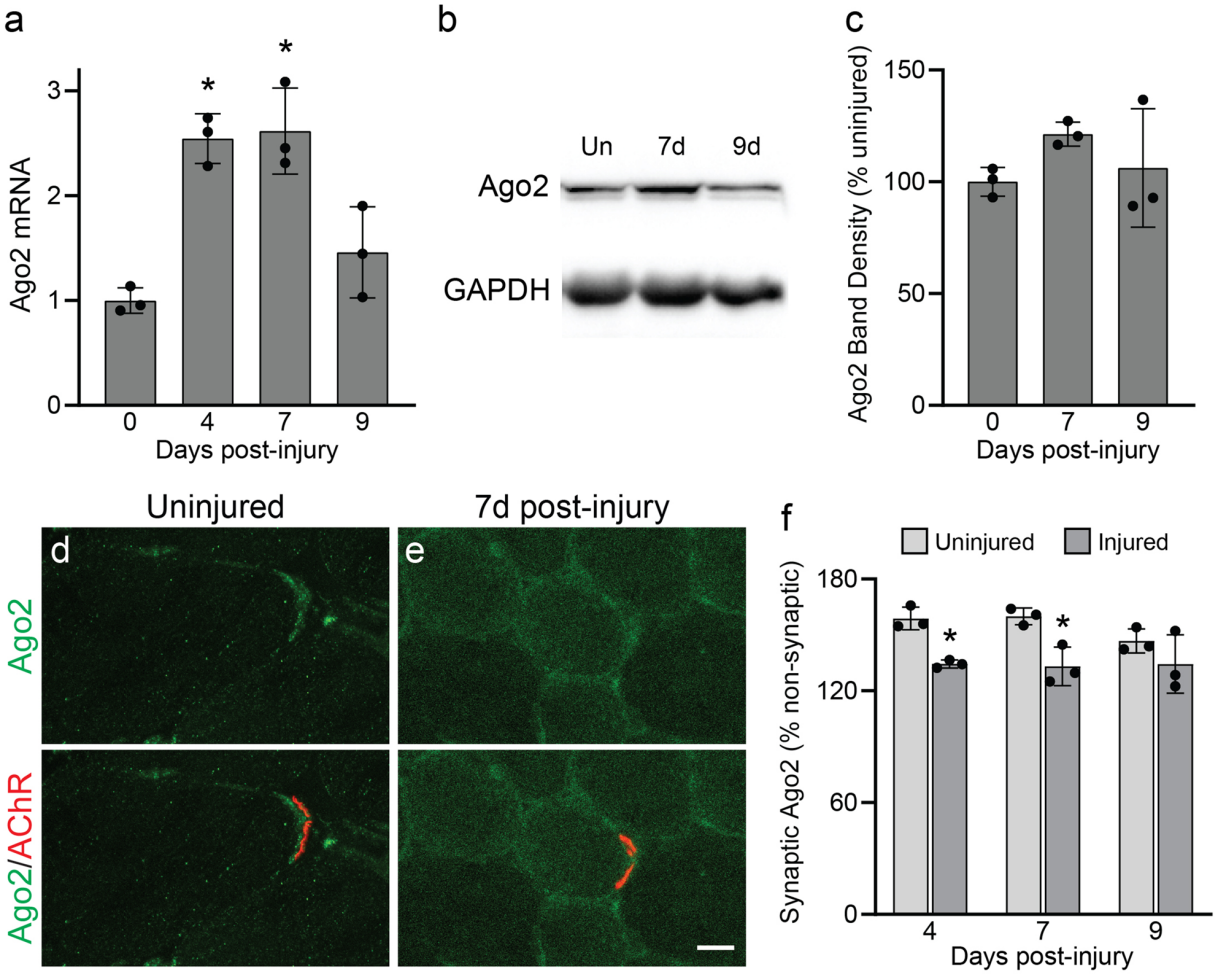
Ago2 expression and distribution is altered in the TA following fibular nerve crush injury. (**a**) qPCR analysis of Ago2 mRNA expression. (**b**,**c**) Western blot analysis of Ago2 protein levels. Images are cropped from the full-length blot, available in Fig. S6. (**d**,**e**) Representative images of Ago2 IHC in TA cross sections collected from uninjured leg (**e**) and at 7 days post-injury. (**f**) Quantification of synaptic Ago2 pixel intensity at 4, 7, and 9 days post-injury, relative to non-synaptic Ago2 pixel intensity. *p < 0.05 versus uninjured leg, One-way ANOVA with Bonferroni post-hoc (**a**,**b**) or unpaired 2-sided t test (**d**). All values reported as mean ± SD; n = 3. Scale bar = 10 µm.

We next assessed whether Ago2 expression and synaptic distribution respond to NMJ denervation. To do so, we crushed the fibular nerve at its intersection with the gastrocnemius tendon, near the knee, and assessed Ago2 gene expression in the TA muscle at 4, 7 and 9 days post-injury. In this injury paradigm, TA muscles are completely denervated at 4 days post-injury, regenerating motor axons make initial contact with endplates at 7 days post-injury, and synaptic refinement to reform fully functional NMJs is underway at 9 days post-injury^19^. We found that Ago2 mRNA levels are upregulated in completely denervated TAs (4 days post-injury) and as motor axons make initial contact with the TA (7 days post-injury) but return to baseline levels at 9 days post-injury as NMJs become reinnervated (Fig. 3a). A similar trend in mRNA expression was observed in the miRNA biogenesis genes DGCR8, Exportin-5 and Dicer (Fig. S2a). Surprisingly, this trend was not observed for Ago2 protein, which remained unchanged at 7 and 9 days post-injury (Fig. 3b,c). Analysis of synaptic Ago2 localization with IHC in TA cross sections revealed that Ago2 is more evenly distributed between synaptic and non-synaptic regions of the muscle fiber at 4 and 7 days post-injury, when NMJs are completely or mostly denervated (Fig. 3d–f). As NMJs begin to be reinnervated at 9 days post-injury, synaptic Ago2 levels are similar to those seen at 4 and 7 days post-injury, however synaptic Ago2 was unexpectedly lower in the contralateral uninjured TA at this timepoint (Fig. 3f). These results suggest that skeletal muscles respond to denervation by inducing Ago2 transcription and changing its distribution without significantly altering overall Ago2 protein levels.

### Ago2 expression and distribution is altered in SOD1^G93A^ muscle

**Figure 4:**
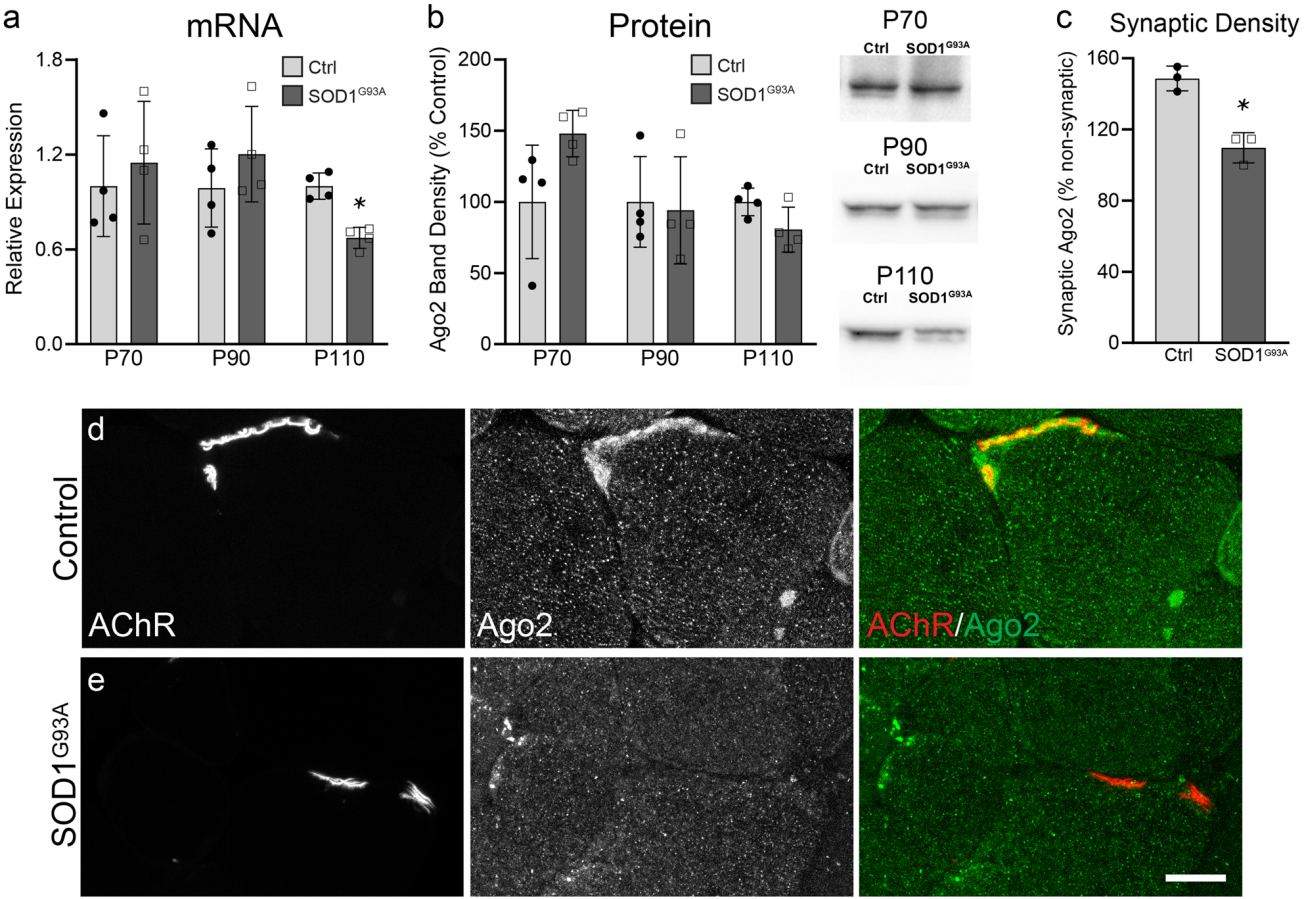
Ago2 expression and distribution are disrupted in SOD1^G93A^ muscle. (**a**) qPCR and (**b**) Western blot analysis of Ago2 levels in TA muscle collected from P70, P90, and P110 SOD1^G93A^ and control littermates (N = 4). mRNA levels are relative to age-matched controls. Protein levels are normalized to total protein (Fig. S7c,f,i). Images are cropped from the full-length blot, available in Fig. S7. (**c**) Mean synaptic Ago2 pixel intensity in TA muscle fibers of P90 SOD1^G93A^ and control littermates, reported as the ratio of synaptic versus non-synaptic pixel intensity within a muscle fiber (N = 3). (**d**,**e**) Representative images of Ago2 (green) distribution at fBTX-labeled NMJs (red) in TA cross sections of P90 control (**d**) and SOD1^G93A^ (**e**) littermates. *p < 0.05, versus age-matched control, unpaired 2-sided T-test. All values reported as mean ± SD; n = 4 for all panels except C, where n = 3. Scale bar = 10 µm.

Progressive NMJ denervation is an early event in ALS^20^. Several studies have shown that the rate of ALS-associated NMJ loss is impacted by levels of miR-126, miR-133b, and miR-206^12–14^. In light of these and our own observations that healthy skeletal muscles respond to NMJ denervation by upregulating Ago2 mRNA and redistributing ago2 protein (Fig. 3), we asked whether skeletal muscles affected by ALS alter Ago2 expression and distribution. To answer this question, we utilized the SOD1^G93A^ ALS mouse model. Progressive NMJ denervation of predominately fast-twitch hindlimb muscles, such as the TA, is well characterized in SOD1^G93A^ mice, where it initiates pre-symptomatically, near P50, and becomes widespread by P90^13,16,20–25^. We found that Ago2 mRNA and protein levels were unchanged in SOD1^G93A^ versus their wild-type control littermates at pre-symptomatic (P70) and early symptomatic (P90) ages (Fig. 4a,b). By P110, however, Ago2 mRNA levels were decreased (Fig. 4a) yet Ago2 protein levels remain unchanged (Fig. 4b) in SOD1^G93A^ TA muscles. Interestingly, we did not observe a consistent trend in SOD1^G93A^-related changes in mRNA levels among miRNA biogenesis genes, where DGCR8 was unchanged, Exportin-5 was increased and Dicer was decreased at P110 while moderate increases in expression of the RISC loading genes Poly(RC) Binding Protein 1 (PCBP1)^26^ and trans-Activation-Responsive RNA-Binding Protein 2 (TARBP2)^1^ were observed in SOD1^G93A^ muscles (Fig. S2b,c). Analysis of Ago2 distribution in the TA of P90 SOD1^G93A^ mice revealed that synaptic enrichment of Ago2 is lost (Fig. 4c–e). Analysis of Ago2 colocalization with fBTX-labeled AChRs in P90 transgenic Thy1YFP; SOD1^G93A^ mice, in which motor axons are labeled with YFP, revealed that synaptic Ago2 enrichment is not affected by NMJ innervation status, where levels were similar between NMJs with high and low colocalization of YFP-labeled axons and AChRs (Fig. S3d). These results suggest that, despite progressive increases in NMJ denervation (Fig. S3a–c)^16,25^, skeletal muscles do not show a trend in upregulation of Ago2 or miRNA biogenesis genes with disease progression in the SOD1^G93A^ ALS mouse model. Moreover, synaptic enrichment of Ago2 is lost in the early symptomatic stage of the model.

### Skeletal muscle health is minimally impacted by Ago2 knockdown in adult mice

**Figure 5:**
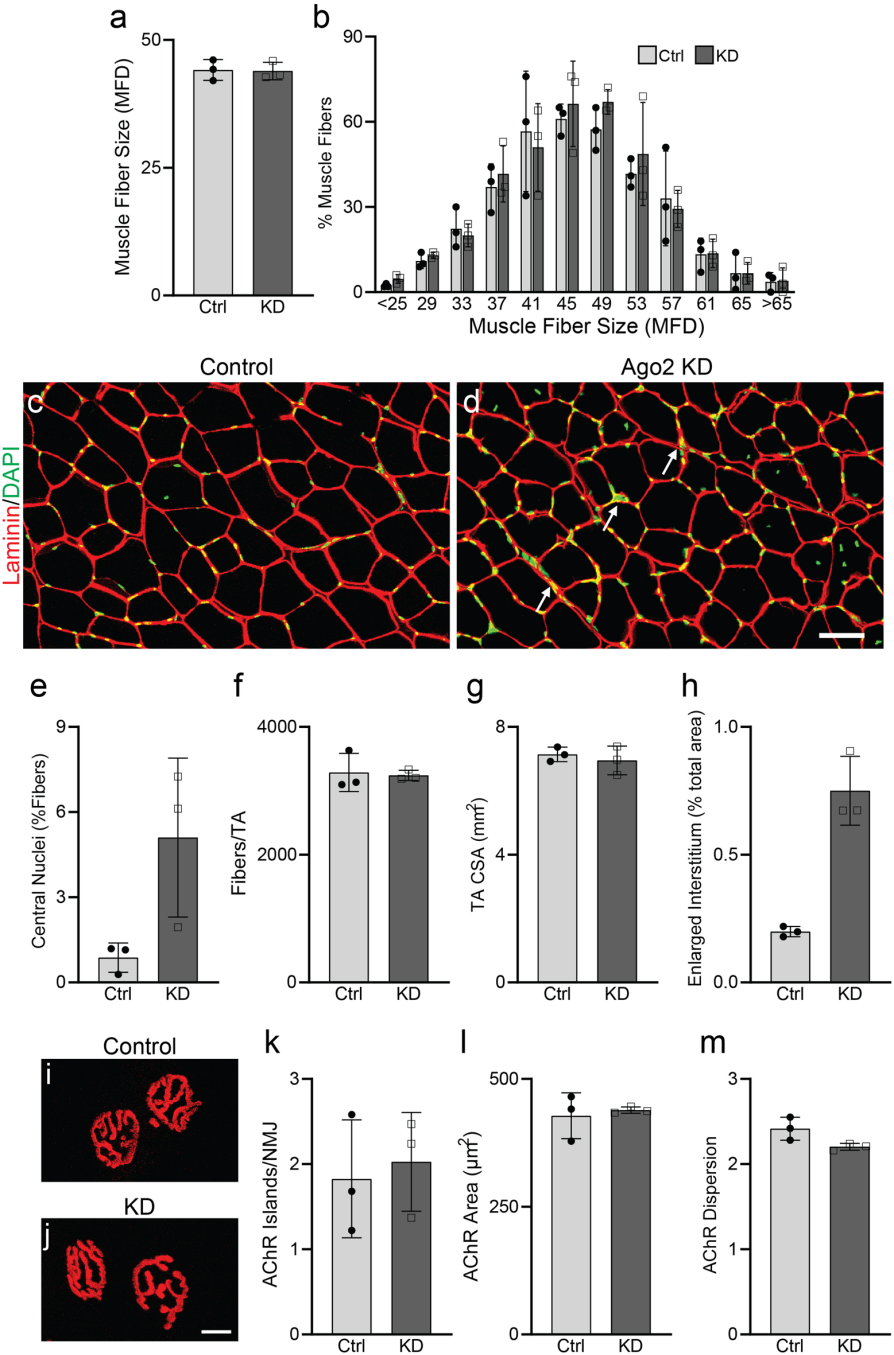
Ago2 knockdown minimally impacts skeletal muscle fiber health in young adult mice. Ago2^fl/fl mice^ received control-eGFP or Cre AAVs to contralateral TAs at P17. (**a**–**h**) Histological analysis of TAs performed at 3 months post-infection. (**a**) Mean muscle fiber size, assessed by measurement of minimum Feret’s diameter (MFD) of muscle fiber cross sections identified by laminin IHC. (**b**) Distribution of TA muscle fiber sizes. (**c**,**d**) Representative images of laminin (red) IHC of TA muscle cross sections collected from control (**c**) and Ago2 KD (**d**) legs. Arrows indicate areas of thickened interstitial space with nuclei. (**e**) Percentage of muscle fibers with centrally located nuclei. (**f**) Number of muscle fibers per TA cross section. (**g**) Cross-sectional area (CSA) of TA muscles. (**h**) Percentage of TA CSA containing thickened interstitial space. (**i**,**j**) Representative images of fBTX-labeled AChR clusters in control (**i**) and Ago2 KD (**j**) EDL muscles. (**k**) Number of AChR islands per NMJ. (**l**) Area of NMJ AChR area. (**m**) AChR cluster dispersion. All values reported as mean ± SD. Unpaired 2-sided t test used for all comparisons except panels (**e**) and (**l**), where unpaired 2-sided t test with Welch’s correction was used, and panel (**h**), where Mann–Whitney test was used. n = 3. Scale bar = 10 µm.

While miRNA-mediated transcriptional regulation is known to play an essential role in skeletal muscles affected by diseases such as ALS, it remains unknown whether Ago2 plays important roles in maintaining healthy adult skeletal muscles. To address this question, we knocked down Ago2 in the TA muscles of juvenile Ago2^fl/fl^ mice^27^ using adeno-associated viruses (AAVs) in which Cre-recombinase (Cre) is expressed under the cytomegalovirus promoter. We injected AAV-Cre or AAV-eGFP (control virus) in contralateral TA muscles of P17 mice. Cre expression in skeletal muscles was confirmed at 3 months post-infection with Cre IHC (Fig. S4a,b). To verify knockdown, Ago2 mRNA levels in the TA were assessed by qPCR at 3 weeks post-infection. Ago2 mRNA levels in AAV-Cre infected TAs were reduced by 75% as compared to contralateral control TAs (Fig. S4c). Continued knockdown of Ago2 protein was observed at 11 weeks post-infection with Ago2 Western blot (Fig. S4d). To evaluate the impact of Ago2 knockdown on the health of young adult skeletal muscles, we collected TA muscles at 3 months post-infection for histological analysis. Measurements of muscle fiber size (Fig. 5a–d), regenerating muscle fibers, indicated by the presence of a nucleus located at muscle fiber center (Fig. 5e), muscle fiber numbers (Fig. 5f) and overall TA muscle size (Fig. 5g) were similar between control and Ago2 KD legs. However, we consistently observed an increased presence of thickened interstitial space with elevated numbers of interstitial nuclei in Ago2 KD versus control TA muscles (Fig. 5h). To determine the impact of Ago2 KD on the structural integrity of the NMJ postsynaptic site, we used fBTX to label AChRs in AAV-Cre- and AAV-eGFP infected EDL muscles. We found that the average number of unique AChR islands, the area occupied by AChR clusters per NMJ, and the dispersion of AChRs to be unchanged in muscles infected with AAV-Cre (Fig. 5i–m).

## Discussion

In this study we examined the expression patterns of Ago2 and miRNA biogenesis genes in conditions in which miRNAs are known to play an essential role in skeletal muscles, namely developmental myogenesis^9,10^, NMJ denervation^13,14^ and ALS-associated progressive NMJ degeneration^12–14^. We show that expression of these genes follows overall trends in miRNA expression patterns in healthy muscle, whereby Ago2 and miRNA biogenesis genes are highly upregulated in developing skeletal muscle and in response to nerve injury in adult skeletal muscle. We also show that Ago2 is diffusely distributed in developing skeletal muscles and becomes localized to postsynapses in adult skeletal muscles after NMJ maturation is completed. Experimental denervation of muscles reverses this pattern of distribution, suggesting that Ago2 distribution within a skeletal muscle fiber is linked to its innervation status. Despite the impact of miRNAs on NMJ health in ALS mouse models^12,14^, we did not observe a consistent upregulation of Ago2 or miRNA biogenesis genes in symptomatic SOD1^G93A^ ALS muscle. Additionally, knocking down Ago2 caused an increased presence of thickened interstitial space with mononucleated cells in skeletal muscles but did not affect muscle fiber size in otherwise healthy young adult mice. Together, our results provide insight on how Ago2, and other genes that carry out the important functions of miRNAs, are utilized by healthy and diseased skeletal muscles.

An unresolved question that arises from our findings is why do skeletal muscles alter Ago2 expression and distribution during development, injury and disease? Increases in Ago2 expression mirror increases in miRNA expression that have been previously observed in development^9^ and following nerve injury^13,14^. This suggests that skeletal muscles upregulate Ago2 to accommodate increased demand for RISC-mediated post-transcriptional gene regulation. However this does not appear to be the case in the TA of symptomatic SOD1^G93A^ mice, in which global increases in miRNAs have been observed^15^. We observed significantly lower Ago2 mRNA and no change in Ago2 protein in the TA muscles of symptomatic (p110) SOD1^G93A^ mice. Thus, the lack of sufficient Ago2 in muscles and specifically at NMJs may impair the reparative actions of miRNAs in progressive neurodegenerative diseases such as ALS.

Previous work demonstrated that Ago2 protein levels are tightly linked to miRNA abundance, where Ago2 becomes increasingly unstable as miRNA levels decrease^28,29^. Experimental depletion of miRNAs causes rapid loss of Ago2 protein without impacting Ago2 mRNA levels^28^. This dependence of Ago2 protein stability on miRNAs may help explain the disparity in Ago2 mRNA and protein levels that we observed in muscles collected from denervated and SOD1^G93A^ muscles. For example, global increases in miRNA levels in SOD1^G93A^ muscles^15^ could explain the relatively moderate decline in Ago2 protein, as compared to Ago2 mRNA, that we observed in TA muscles of p110 SOD1^G93A^ mice. It may also explain the increase in synaptic Ago2 distribution that we observed in healthy adult skeletal muscles. Given the known roles of muscle-specific miRNAs in NMJ maintenance^13,14^, it is possible that synaptic nuclei maintain elevated miRNA levels which, in turn, increase the stability of Ago2 protein located in close proximity to NMJs. Along these lines, depressed miRNA expression by extrasynaptic nuclei would decrease Ago2 protein stability, leading to lower levels of Ago2 protein in extrasynaptic regions of the muscle.

Our findings suggest that synaptic enrichment of Ago2 in skeletal muscles is linked to NMJ innervation status. We found that Ago2 is evenly distributed between synaptic and non-synaptic regions of skeletal muscle fibers that lack mature NMJs, in early development, or have a preponderance of denervated NMJs, following nerve injury and in SOD1^G93A^ muscles. By contrast, Ago2 distribution is roughly 50% higher in synaptic versus non-synaptic regions of healthy adult muscle fibers with intact NMJs. As mentioned above, these changes could be linked to miRNA expression in synaptic versus non-synaptic nuclei. It is also possible that Ago2 gene expression is confined to synaptic nuclei in healthy adult muscles while non-synaptic nuclei upregulate Ago2 expression in developing or injured skeletal muscles. This would not only lead to increases in overall levels of Ago2, it would account for the similar levels of synaptic and non-synaptic Ago2 that we observed in developing and injured skeletal muscles. Another possibility is that Ago2 is tethered to the synaptic region by an unknown mechanism which is linked to the presence of motor axon-derived molecules. In this case retraction of motor axons from the endplate would lead to loss of these molecules and uncoupling of Ago2.

Although our analysis of orthogonal projections of confocal images shows Ago2 highly enriched in the postsynaptic region in adult NMJs, we cannot exclude the possibility that Ago2 is also present in motor axon terminals. Both Ago2 and Dicer have been observed in motor axons of the sciatic nerve while, similar to our findings, Ago2 was observed at mouse NMJs and displayed some colocalization with the neurofilament heavy chain motor axon marker in 3D image reconstructions^30^. The recent evidence that ALS-related mutations disrupt RISC activity in motor neurons and their axons^26,30–33^ warrants further exploration of the precise roles of Ago2 at the NMJ presynapse. In addition to motor axon terminals, Ago2 is likely to be present in perisynaptic Schwann cells (PSCs), the glial cells that envelop the NMJ. In fact, transcriptomic analysis of PSCs that were FACS-isolated from juvenile mice has provided evidence that Ago2 is expressed by these cells^34^. In the current study we did not label PSCs in our Ago2 IHC experiments and so their contribution to synaptic Ago2 localization is not clear.

Using AAV-mediated expression of Cre-recombinase to temporally knockdown Ago2 in skeletal muscles of juvenile mice, we found that reduced levels of Ago2 caused an increased presence of thickened interstitial space, moderately altered the morphology of AChR clusters, but did not impact the size of muscle fibers or their NMJ endplates. The absence of an overtly deleterious phenotype following Ago2 knockdown is in line with previous studies that found that otherwise healthy skeletal muscles are minimally impacted by deletion of individual miRNAs^13,35,36^. For example, global deletion of miR-133b did not produce an observable effect on the health of juvenile or adult skeletal muscle fibers, their NMJs or their transcriptome^36^. By contrast, deletion of miR-133b in the *mdx* mouse model of Duchenne muscular dystrophy exacerbated dystrophic muscle pathology and induced widespread transcriptomic changes^36^. These findings suggest that miRNAs, and Ago2, may serve as emergency responders that largely remain on standby in healthy skeletal muscles. In support of this, Ago2 has been shown to be mostly associated with low molecular weight RISC, devoid of mRNA, in healthy skeletal muscles^37^. However, it is possible that redundant levels of Ago2 or compensatory Ago2 translation may contribute to the absence of a clear phenotype in our Ago2 knockdown model. Determining the extent to which the actions of Ago2 in skeletal muscles impact their development or the progression of NMJ deterioration in ALS, and whether its function as an RNA-binding protein becomes compromised in ALS will provide much needed insight on the role of miRNAs in skeletal muscles.

## Materials and methods

### Animals

SOD1^G93A^ (RRID:IMSR_JAX:004435^38^) and Thy1-YFP16 (RRID:IMSR_JAX:003709^39^) mice were purchased from The Jackson Laboratory. All comparisons involving SOD1^G93A^ mice were made between SOD1^G93A^ and WT littermates. Ago2^fl/fl^ mice (RRID:IMSR_JAX:016520^27^) were a generous gift from Arul Chinnaiyan. Male and female mice were used for all studies. Mice were maintained on a mixed genetic background, appeared healthy and were not subjected to non-related procedures prior to the study. Sample size determination was based on previous gene expression and IHC studies in skeletal muscles performed by our laboratory^19,22,36^ with the goal of minimizing research animal use and detecting robust effects; a power analysis was not used. Ninety-four mice were used for this study. No a priori inclusion/exclusion criteria were established, however no animals were excluded from this study. Blinding was not used at any experimental stage. Mice were transported out of the housing facility for surgeries and tissue collection. Mice were anesthetized by intraperitoneal injection of Ketamine (50 mg/kg) and xylazine (7.5 mg/kg) and administered ophthalmic ointment for fibular nerve crush surgeries or administration of AAVs. Buprenorphine was administered to mice prior to fibular nerve crush. Arousal and respiration were monitored throughout procedures and mice were monitored for pain and administered buprenorphine as needed for 48 h post-surgery. Mouse pups were sacrificed by decapitation following isoflurane anesthesia. A lethal dose of isoflurane was administered to sacrifice adult mice. Animals were provided water and food ad libitum and housed in a 12 h light/dark cycle. All surgeries and tissue collection were performed during daylight hours. Breeding, housing, and experimental use of animals were performed in a pathogen free environment in accordance with the National Institutes of Health and Brown University Institutional Animal Care and Use Committee (Protocol# 19-05-0013) guidelines. All animal experiments performed in this study were approved by the Institutional Animal Care and Use Committee at Brown University and are reported in accordance with ARRIVE guidelines.

### Immunohistochemistry

Antibodies used for immunohistochemistry (IHC) include: rabbit anti-Ago2 (1:250; Abcam no. 32381, RRID:AB_867543), mouse anti-synaptotagmin-2 (1:50; DSHB no. znp-1, RRID:AB_531910), rabbit anti-Laminin (1:250, Sigma-Aldrich #L9393, RRID:AB_477163), Alexa Fluor 488 conjugated polyclonal goat anti-rabbit antibody (1:1000, Invitrogen no. A-11008, RRID:AB_143165), Alexa Fluor 568 conjugated polyclonal goat anti-rabbit antibody (1:1000, Invitrogen no. A11036, RRID:AB_10563566) and Alexa Fluor 647 conjugated goat anti-mouse IgG1 antibody (1:1000, Invitrogen no. A-21240, RRID:AB_141658). Alexa Fluor 555 conjugated alpha-bungarotoxin (fBTX, 1:1000 no. B35451, RRID:AB_2617152, Invitrogen, Carlsbad, CA, USA) and Alexa Fluor 488 conjugated alpha-bungarotoxin (1:1000 no. B13422, Invitrogen, Carlsbad, CA, USA) were used to label AChRs.

Muscles were dissected from mice following transcardial perfusion with 4% paraformaldehyde. For IHC of whole mounted muscles, dissected muscles were incubated in blocking buffer (5% BSA, 3% goat serum, 0.5% Triton X-100 in PBS) for 2 h at room temperature, incubated in primary antibody diluted in blocking buffer for 48 h at 4 °C, washed 5 min in PBS 3 times, incubated in secondary antibody and fBTX for 2 h at room temperature, washed 5 min in PBS 3 times and mounted to microscope slides in Vectacshield (Vector Laboratories, Burlingame, CA, USA). For IHC of muscle cross-sections, muscles were incubated in 30% sucrose in PBS for 48 h at 4 °C, embedded in Tissue Freezing Medium (General Data no TFM-5, Cincinnati, OH), and cross-sectioned at 16 µm with a cryostat. Muscle cross sections were placed on gelatin-coated glass microscope slides, washed 3 times with PBS, incubated in blocking buffer (5% BSA, 3% goat serum, 0.1% Triton X-100 in PBS) for 1 h at room temperature, incubated in primary antibody overnight at 4 °C, washed 3 times with PBS, incubated in secondary antibody and fBTX for 1 h at room temperature, washed 3 times with PBS, and covered in Vectashield before coverslip application. Muscle cross-sections used for laminin IHC were stained with DAPI (1:1000 in PBS) for 20 min at room temperature and washed 2 times with PBS prior to Vectashield application.

### Image acquisition

Imaging was performed with a Zeiss LSM 710 laser scanning confocal microscope (Carl Zeiss Microscopy, Berlin, Germany) using a 20× (0.8 numerical aperture) objective or 40× (1.3 NA) oil-immersion objective. Zeiss Zen software (RRID: SCR_013672) was used for maximum intensity projections and stitching of tile scans.

### Image analysis

Ago2 pixel intensity analysis was performed on 16 µm cross-sections collected from the medial TA following Ago2/fBTX IHC. Ago2 pixel intensity measurements were performed using Zeiss Zen software where the synaptic region of the muscle fiber was identified by fBTX labeling. Ago2 pixel intensity measurements were performed on all muscle fibers within a cross-section that displayed an fBTX^+^ synaptic region. At least 50 muscle fibers per animal were analyzed.

Muscle fiber size, TA size, centralized nuclei count and enlarged interstitial space measurements were performed on 16 µm cross-sections collected at 0.3 mm intervals from the medial TA using laminin/DAPI IHC. Muscle fiber size was determined by measuring the minimum Feret’s diameter of laminin^+^ outlines of muscle fiber cross-sections. At least 300 muscle fibers per animal were sampled using the grid function in ImageJ. Overall TA size was calculated by measuring the area of 2–4 cross-sections collected from the medial TA. This measurement was performed on tile scan confocal images of entire TA cross-sections following laminin IHC. The number of muscle fibers per TA muscle was approximated by dividing the cross-sectional area of the TA muscle by the average muscle fiber cross-sectional area following laminin IHC. Enlarged interstitial areas were identified by the presence of thickened laminin^+^ regions between muscle fibers that contained at least one nucleus that was not immediately adjacent to a muscle fiber, as described previously^36^. Cross-sectional area and minimum Feret’s diameter measurements were performed with ImageJ (RRID:SCR_003070).

Post-synaptic NMJ analysis was performed on whole mounted EDL muscle following fBTX labeling of AChRs. To determine the number of AChR islands per NMJ, the number of discrete, non-touching fBTX^+^ islands were counted. AChR area was determined by measuring the area of fBTX^+^ pixels at a given NMJ following signal thresholding using ImageJ^40^. AChR dispersion was calculated by dividing the total endplate area, inclusive of fBTX^+^ and fBTX^−^ pixels, by the area of fBTX^+^ pixels for a given NMJ. At least 50 NMJs per animal were analyzed and NMJs were sampled from tiled confocal images of the EDL muscle using the grid function in ImageJ. Area measurements were performed with ImageJ.

Ago2-AChR and axon-AChR colocalization analyses were performed on maximum intensity projection images obtained from 16 µm TA cross-sections collected from P90 Thy1-YFP and SOD1^G93A^; Thy1-YFP mice following Ago2 and fBTX IHC. Weighted colocalization coefficients were measured with Zeiss Zen Black software (RRID: SCR_013672).

### RNA extraction and quantitative RT-PCR

Total RNA was isolated from muscles using Trizol (Life Technologies) and the Aurum Total RNA Mini Kit (Bio-Rad). RNA was reverse transcribed using iScript Reverse Transcription Supermix (Bio-Rad). qPCR was performed with iTAQ SYBR Green Supermix (Bio-Rad) containing 300 nM forward and reverse primers using the CFX Connect Real Time PCR System (Bio-Rad). Cycling parameters were 95 °C for 30 s, 40 cycles of 95 °C (5 s) and 58 °C (30 s), followed by a melt curve consisting of 5 s 0.5 °C incremental increases ranging from 65 to 95 °C. Primer sequences used in this study are listed in Table 1.

**Table 1:** Sequences of primers used for qPCR.

Gene	Forward primer (5′–3′)	Reverse primer (5′–3′)
Ago2	ATGGACATCCCCAAAATTGA	TAAAGTGCTGGACCATGTGC
DGCR8	GAAGAACTGGAGTATTTTAACCACA	AGCCTTGCTTGTCAGCTCAT
Dicer	GTGCTGCAGTAAGCTGTGCTA	AGAAAGGACCCATTGGTGAGG
Exportin-5	GTGTCGAAGAAGACTGCCGA	GTGCAGACATCCTCGTGCTT
GAPDH	CCCACTCTTCCACCTTCGATG	GTCCACCACCCTGTTGCTGTAG
PCBP1	GGTGCAGCTAGAACAGCGTA	GAAACAGCAGGAAGGGGGTT
pri-miR-133b	GCTGGTCAAACGGAACCAAG	ATATTGAGCTTTGCCAGCCCT
pri-miR-206	GGCCACATGCTTCTTTATATCC	AAACCACACACTTCCTTACATTCC
TARBP2	GCACTACTACAGGCTGCGGG	GGTCGTACACGGGCGTCTTT

### Whole cell lysate collection and western blotting

Flash frozen muscle was grinded into a fine powder over liquid nitrogen and homogenized in 2% SDS cell lysis buffer containing protease inhibitors (Thermo #78430) and sodium orthovanadate. Protein concentrations of whole cell lysate were obtained with the Bradford assay. Whole cell lysate was diluted in laemmli buffer and denatured at 95 °C for 10 min prior to electrophoresis on a 10% SDS-PAGE gel. Samples were transferred to nitrocellulose membrane using wet transfer, blocked for 1 h at room temperature in blocking solution (5% nonfat milk diluted in 0.1% Tween Tris buffer solution), incubated overnight at 4 °C in anti-glyceraldehyde-3-phosphate dehydrogenase (GAPDH, 1:15,000, Rockland Antibodies #600–401-A33, RRID:AB_2107593) or anti-Ago2 (1:500, Abcam #32381, RRID:AB_867543) diluted in blocking solution, washed 4 times with 0.1% Tween Tris buffer solution, incubated 1 h at room temperature in peroxidase labeled secondary antibody (1:5000, Ago2; 1:15,000, GAPDH) and washed 4 times with 0.1% Tween Tris buffer solution. Blots were visualized with ECL reagent (GE Healthcare) and a ChemiDoc Imager (Bio-Rad). Ago2 band density was normalized as a ratio of GAPDH band density or total protein, using Coomassie staining.

### Fibular nerve crush injury

Fibular nerve crush surgeries were performed as described previously^19^. Briefly, following administration of anesthesia and analgesic, a small incision was made in the hindleg to access the nerve crush site. The common fibular nerve was crushed at the point where it crosses the gastrocnemius tendon with small forceps for 5 s. Stitches were applied to the incision site. Mice were monitored after surgery and buprenorphine was administered as needed. Comparisons of Ago2 localization were made between contralateral injured and uninjured legs. Comparisons of gene expression were made between muscles of injured and uninjured mice where randomization of treatments was not used.

### Adeno-associated virus (AAV) administration

AAVs were delivered to the belly of TA muscles of anesthetized mice via intramuscular injection (50 µL/muscle, diluted in sterile saline) using a 31-gauge needle. AAVs used in this study include AAV9.CMV.Pl.Cre.rBG (3.3 × 10^9^ GC/leg; Addgene no. 105537-AAV9) and AAV1.CMV.PI.eGFP.WPRE.bGH ((3.3 × 10^9^ GC/leg; Addgene #105530-AAV1).

### Statistics

Distribution normality of residuals was assessed by Shapiro–Wilk test and assessment of QQ plots. Non-parametric tests are reported for data with non-normal distribution of residuals while parametric tests are reported for data with normal distribution of residuals. However, given the inherent uncertainty in assessing the normality of data with low sample sizes^41^, we performed alternate tests for all comparisons (Table 2). These include Student’s unpaired t-test, with Welch’s correction as necessary, and Mann–Whitney test to compare 2 means and ordinary one-way ANOVA with Bonferroni post hoc and Kruskal–Wallis ANOVA to compare 3 or more means. Statistical tests for each experiment are described in the figure legends. For all experiments, n indicates the number of animals. Data are expressed as mean ± standard deviation. Statistical analyses performed with GraphPad Prism (RRID: SCR_002798) software. Results of normality, parametric, and non-parametric tests are provided in Table 2.

**Table 2:** Summary of statistics.

Figure	Dependent variable	Independent variable	Shapiro–Wilk P value (respectively)	Parametric test	P value	Non-parametric test	P value
1a	pri-miR-133b	Age (P1, P6, P21, Adult)	0.4284, 0.5661, 0.0007, 0.0315	One Way ANOVA	0.0003	Kruskal–Wallis	0.0003
1a	pri-miR-206	Age (P1, P6, P21, Adult)	0.4938, 0.9995, 0.5775, 0.8833	One Way ANOVA	0.0002	Kruskal–Wallis	< 0.0001
1b	Ago2 mRNA	Age (P1, P6, P21, Adult)	0.9494, 0.8547, 0.9761, 0.9951	One Way ANOVA	0.0002	Kruskal–Wallis	0.0014
1d	Ago2 protein	Age (P1, P6, P21, Adult)	0.1728, 0.8973, 0.1342, 0.3403	One Way ANOVA	0.0151	Kruskal–Wallis	0.0118
1e	DGCR8 mRNA	Age (P1, P6, P21, Adult)	0.6369, 0.2530, 0.3631, N/A	One Way ANOVA	< 0.0001	Kruskal–Wallis	0.0008
1e	Exportin-5 mRNA	Age (P1, P6, P21, Adult)	0.9521, 0.9999, 0.2983, 0.2983	One Way ANOVA	< 0.0001	Kruskal–Wallis	0.0064
1e	Dicer mRNA	Age (P1, P6, P21, Adult)	0.9999, 0.9162, 0.9932, 0.9356	One Way ANOVA	< 0.0001	Kruskal–Wallis	0.0007
2f	Synaptic Ago2	Age (P1, P21, Adult)	0.9809, 0.8061, 0.8621	One Way ANOVA	0.053	Kruskal–Wallis	0.0608
3a	Ago2 mRNA	Days P.I. (0,4,7,9)	0.3872, 0.5546, 0.3330, 0.9420	One Way ANOVA	0.0006	Kruskal–Wallis	0.0039
3c	Ago2 protein	Days P.I. (0,7,9)	0.7077, 0.6960, 0.1307	One Way ANOVA	0.3148	Kruskal–Wallis	0.3607
3f	Synaptic Ago2	4 days injured vs uninjured	0.1884, 0.9231	T-test	0.0028	Mann–Whitney	0.1
3f	Synaptic Ago2	7 days injured vs uninjured	0.6688, 0.4306	T-test	0.0147	Mann–Whitney	0.1
3f	Synaptic Ago2	9 days injured vs uninjured	0.2393, 0.3684	T-test	0.2728	Mann–Whitney	0.4
4a	Ago2 mRNA	P70 control vs SOD1G93A	0.1595, 0.9435	T-test	0.5783	Mann–Whitney	0.6857
4a	Ago2 mRNA	P90 control vs SOD1G93A	0.8770, 0.2587	T-test	0.3313	Mann–Whitney	0.4857
4a	Ago2 mRNA	P110 control vs SOD1G93A	0.3669, 0.4679	T-test	0.0008	Mann–Whitney	0.0286
4b	Ago2 protein	P70 control vs SOD1G93A	0.0729, 0.9073	T-test	0.0673	Mann–Whitney	0.0571
4b	Ago2 protein	P90 control vs SOD1G93A	0.1411, 0.2632	T-test	0.1556	Mann–Whitney	0.6857
4b	Ago2 protein	P110 control vs SOD1G93A	0.8817, 0.3284	T-test	0.0803	Mann–Whitney	0.2
4c	Synaptic Ago2	P90 control vs SOD1G93A	0.8247, N/A	T-test	0.0035	Mann–Whitney	0.1
5a	Muscle fiber size	Control vs Cre AAV	0.8939, 0.2513	T-test	0.9009	Mann–Whitney	> 0.9999
5b	< 25 µm MFD	Control vs Cre AAV	N/A, 0.6369	T-test	0.0686	Mann–Whitney	0.2
5b	29 µm MFD	Control vs Cre AAV	0.3631, > 0.9999	T-test	0.2879	Mann–Whitney	0.5
5b	33 µm MFD	Control vs Cre AAV	0.6878, > 0.9999	T-test	0.6458	Mann–Whitney	0.8
5b	37 µm MFD	Control vs Cre AAV	0.5928, 0.1939	T-test	0.5626	Mann–Whitney	> 0.9999
5b	41 µm MFD	Control vs Cre AAV	0.7391, 0.5665	T-test	0.7269	Mann–Whitney	0.8
5b	45 µm MFD	Control vs Cre AAV	0.3631, 0.1270	T-test	0.5935	Mann–Whitney	0.7
5b	49 µm MFD	Control vs Cre AAV	0.9265, 0.2196	T-test	0.126	Mann–Whitney	0.3
5b	53 µm MFD	Control vs Cre AAV	0.7804, 0.4778	T-test	0.5553	Mann–Whitney	> 0.9999
5b	57 µm MFD	Control vs Cre AAV	0.7017, 0.9152	T-test	0.741	Mann–Whitney	> 0.9999
5b	61 µm MFD	Control vs Cre AAV	0.5098, 0.7804	T-test	0.943	Mann–Whitney	> 0.9999
5b	65 µm MFD	Control vs Cre AAV	0.5827, 0.2530	T-test	> 0.9999	Mann–Whitney	> 0.9999
5b	> 65 µm MFD	Control vs Cre AAV	0.2983, 0.9643	T-test	0.9228	Mann–Whitney	> 0.9999
5e	Central nuclei	Control vs Cre AAV	0.0743, 0.3884	Welch's T-test	0.1156	Mann–Whitney	0.1
5f	Fibers/TA	Control vs Cre AAV	0.1181, 0.1321	T-test	0.803	Mann–Whitney	0.7
5g	TA CSA	Control vs Cre AAV	0.9279, 0.9203	T-test	0.5497	Mann–Whitney	> 0.9999
5h	Enl. Interstitium	Control vs Cre AAV	0.9641, 0.0085	Welch's T-test	0.0175	Mann–Whitney	0.1
5k	AChR Islands/NMJ	Control vs Cre AAV	0.6473, 0.3811	T-test	0.7207	Mann–Whitney	> 0.9999
5l	AChR Area	Control vs Cre AAV	0.5163, 0.4633	Welch's T-test	0.714	Mann–Whitney	> 0.9999
5m	AChR dispersion	Control vs Cre AAV	0.9592, 0.7262	T-test	0.0587	Mann–Whitney	0.1
S2a	DGCR8 mRNA	Days P.I. (0,4,7,9)	0.3631, 0.8362, 0.3535, 0.7902	One Way ANOVA	0.0001	Kruskal–Wallis	0.0016
S2a	Exportin-5 mRNA	Days P.I. (0,4,7,9)	0.8296, 0.2327, 0.9434, 0.5367	One Way ANOVA	0.0011	Kruskal–Wallis	0.0014
S2a	Dicer mRNA	Days P.I. (0,4,7,9)	0.4391, 0.9404, 0.7391, 0.8899	One Way ANOVA	0.0009	Kruskal–Wallis	0.0039
S2c	DGCR8 mRNA	P110 control vs SOD1G93A	0.1256, 0.3261	T-test	0.1688	Mann–Whitney	0.2
S2c	Exportin-5 mRNA	P110 control vs SOD1G93A	0.0159, 0.0389	T-test	0.0026	Mann–Whitney	0.0286
S2c	Dicer mRNA	P110 control vs SOD1G93A	0.9760, 0.2418	T-test	0.0026	Mann–Whitney	0.0253
S2d	PCBP1 mRNA	P110 control vs SOD1G93A	0.0276, 0.8698	T-test	0.0615	Mann–Whitney	0.1143
S2d	TARBP2 mRNA	P110 control vs SOD1G93A	0.1830, 0.6007	T-test	0.041	Mann–Whitney	0.1143

## Supplementary Information

Supplementary Figures.

## Data Availability

The datasets generated during the current study are available from the corresponding author on reasonable request.
