# Supplementary material for: Argonaute 2 is lost from neuromuscular junctions affected with amyotrophic lateral sclerosis in SOD1G93A mice
Source: Sci Rep. 2022 Mar 17;12:4630. doi: 10.1038/s41598-022-08455-y (PMC8931107; doi:10.1038/s41598-022-08455-y)
Supplement: Supplementary file 1 — Supplementary Figures. [file 41598_2022_8455_MOESM1_ESM.pdf]

Supplementary Figures

Title: Argonaute 2 is lost from neuromuscular junctions affected by Amyotrophic Lateral Sclerosis in SOD1^{G93A} mice

Authors: Dillon Shapiro[1], Ryan Massopust[2], Thomas Taetzsch[2], Gregorio Valdez[2,3,4]

Supplementary Figure 1

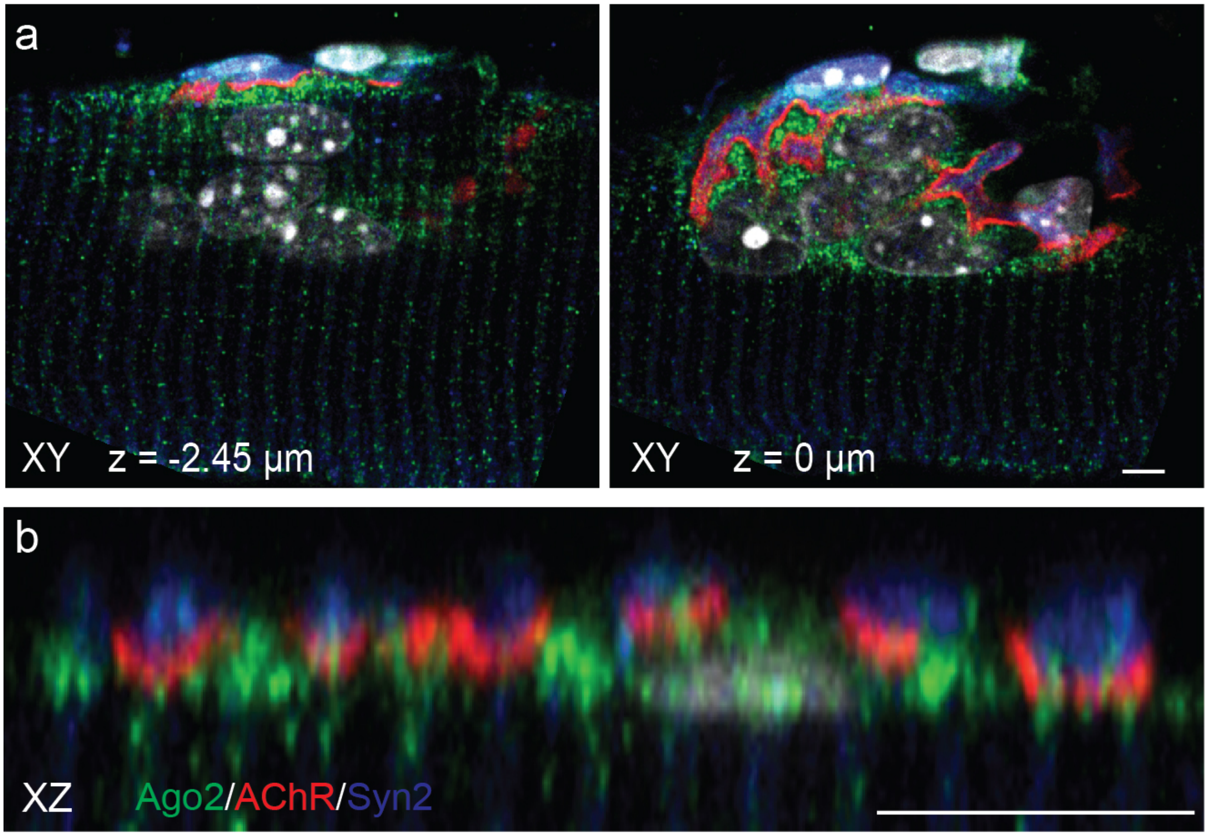

Figure S1. Ago2 is concentrated at the NMJ postsynapse in young adult muscle. (a) Individual optical sections and (b) corresponding orthogonal projection of Ago2 distribution at an NMJ in a young adult EDL muscle. Ago2 (green), Synaptotagmin-2 (blue) and fBTX (red) IHC show that Ago2 intercalates between fBTX labeled AChR clusters of the NMJ postsynapse. Scale bar = 10 µm.

Supplementary Figure 2

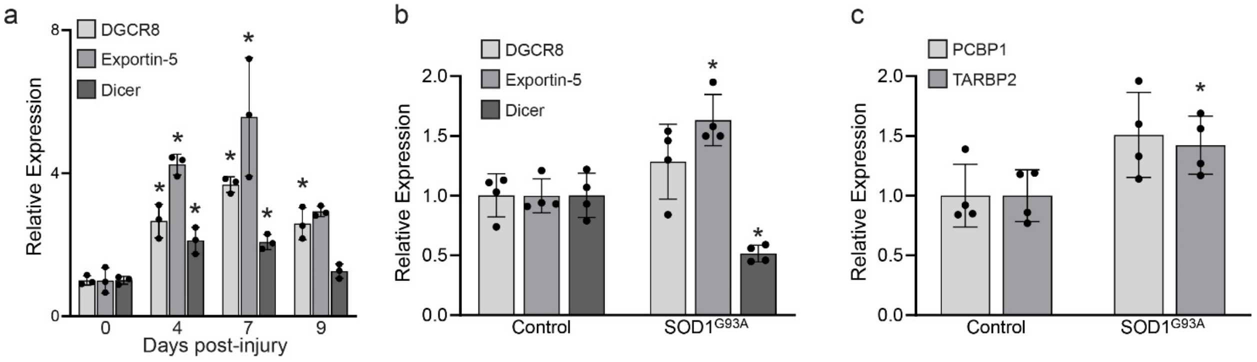

Figure S2. (a) qPCR analysis of miRNA biogenesis genes in TA muscles of young adult mice following fibular nerve crush. (b) Representative images of Coomassie stain used to determine total protein levels in the TA of control (C) and SOD1^{G93A} (S) mice for normalization of Ago2 densitometry analysis in Fig. 4. Full length images of Coomassie stained membranes are available in Fig. S7. (c) qPCR analysis of miRNA biogenesis genes in TA muscles of symptomatic (P110) and (d) early symptomatic (P90) SOD1^{G93A} mice. (N = 4). mRNA levels are relative to age-matched controls. * $p < 0.05$ versus control. Ordinary one-way ANOVA used in panel a. Unpaired 2-sided T-test used for all other comparisons except c and d, where Mann-Whitney test was used to compare Exportin-5 and PCBP1 expression. All values reported as mean ± SD.

Supplementary Figure 3

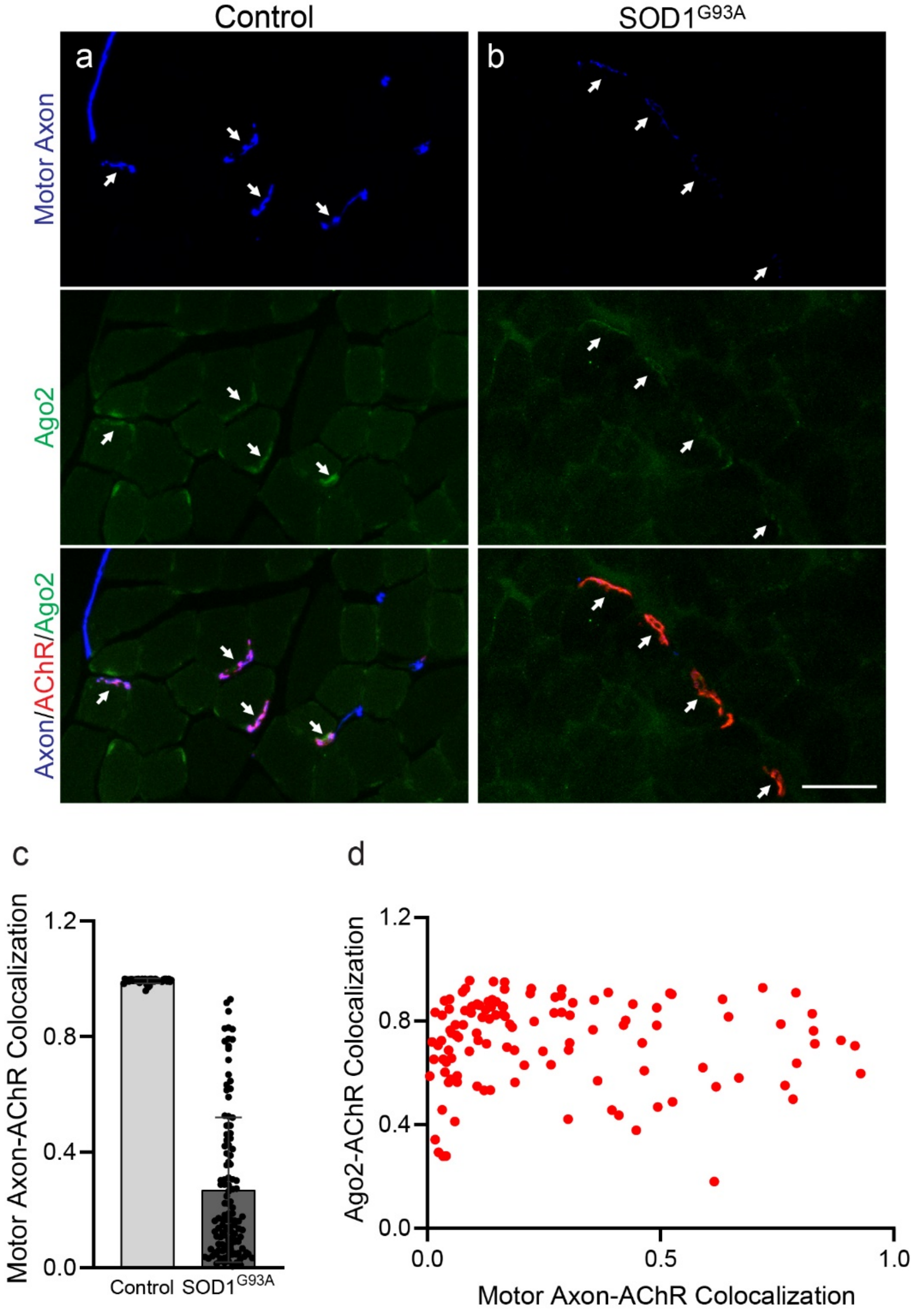

Figure S3. (a-b) Representative images of Ago2 (green) distribution in P90 Thy1-YFP control (a) and SOD1^{G93A};Thy1-YFP (b) TA cross sections. Motor axons are labeled with YFP (blue) and AChRs are labeled with fBTX (red). Arrows denote NMJs. In SOD1^{G93A};Thy1-YFP muscles, synaptic Ago2 enrichment is similar between partially innervated NMJs (top left arrow in panel b) and fully denervated NMJs (bottom right arrow in panel b). (c) TA muscles of P90 SOD1^{G93A};Thy1-YFP mice have increased numbers of NMJs displaying decreased colocalization of YFP labeled motor axon terminals with fBTX labeled AChRs. Values reported as mean ± SD. (d) Analysis of Ago2-AChR versus YFP labeled motor axon-AChR colocalization at a given NMJ in P90 SOD1^{G93A};Thy1YFP TA cross sections. $r = -0.02912$, $p = 0.7542$. Data points represent individual NMJs. Scale bar = 50 µm.

Supplementary Figure 4

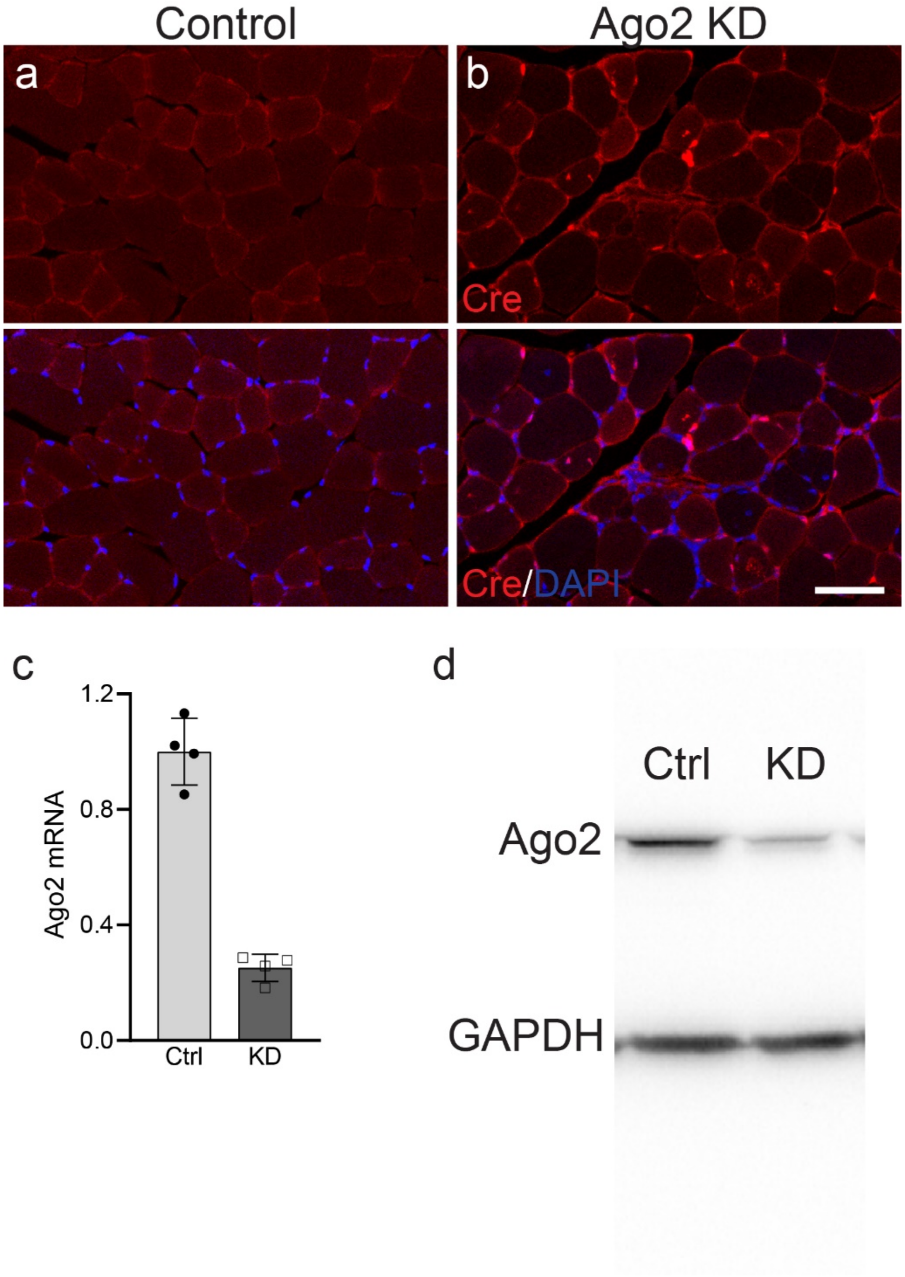

Figure S4. Verification of Ago2 knockdown in Ago2$^{fl/fl}$ TA. (a-b) Cre IHC in cross sections of control (a) and AAV-Cre infected (b) TA cross sections shows increased Cre with localization to myonuclei in skeletal muscle fibers of AAV-Cre infected muscle. (c) qPCR analysis of Ago2 mRNA expression in TAs collected at 3 weeks post-infection. (d) Western blot analysis of Ago2 protein levels in TAs collected at 11 weeks post-infection. Image is cropped from the full-length blot, available in Fig. S5. Scale bar = 50 µm.

Supplementary Figure 5

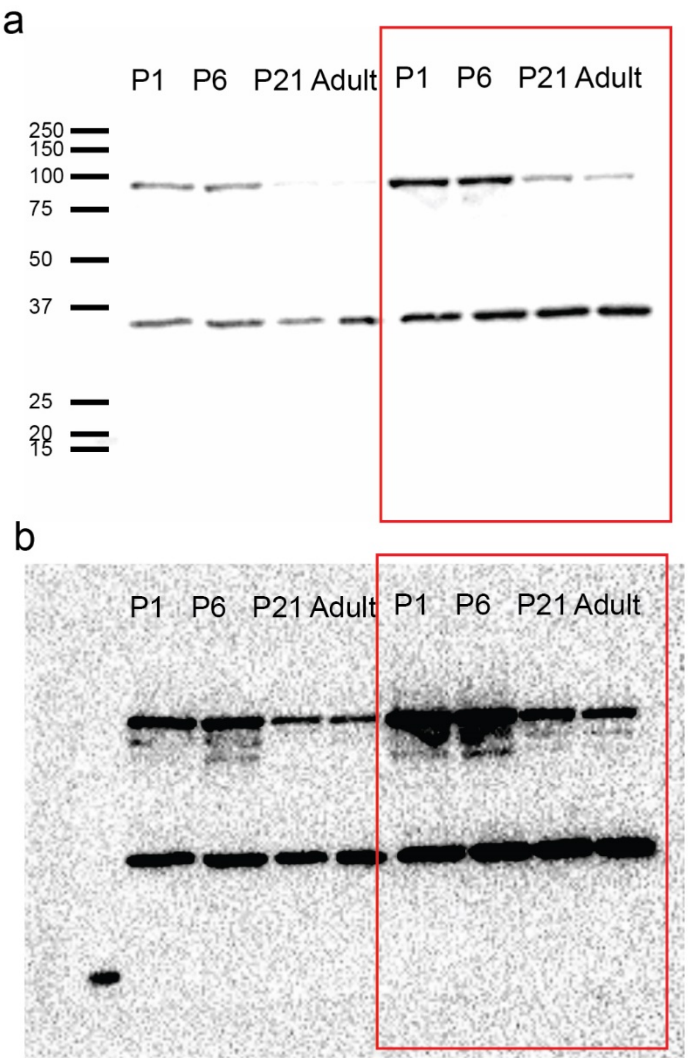

Figure S5. Uncropped images with protein size markers of Ago2/GAPDH Western blot of developmental TA muscles displayed in Figure 1. (a) Original scan without changes to brightness and contrast. (b) Brightness and contrast altered to show membrane edges. Membranes were cut and probed separately for Ago2 (top) and GAPDH (bottom), then placed together for imaging. Red boxes indicate the cropped portion of the membrane displayed in figure 1.

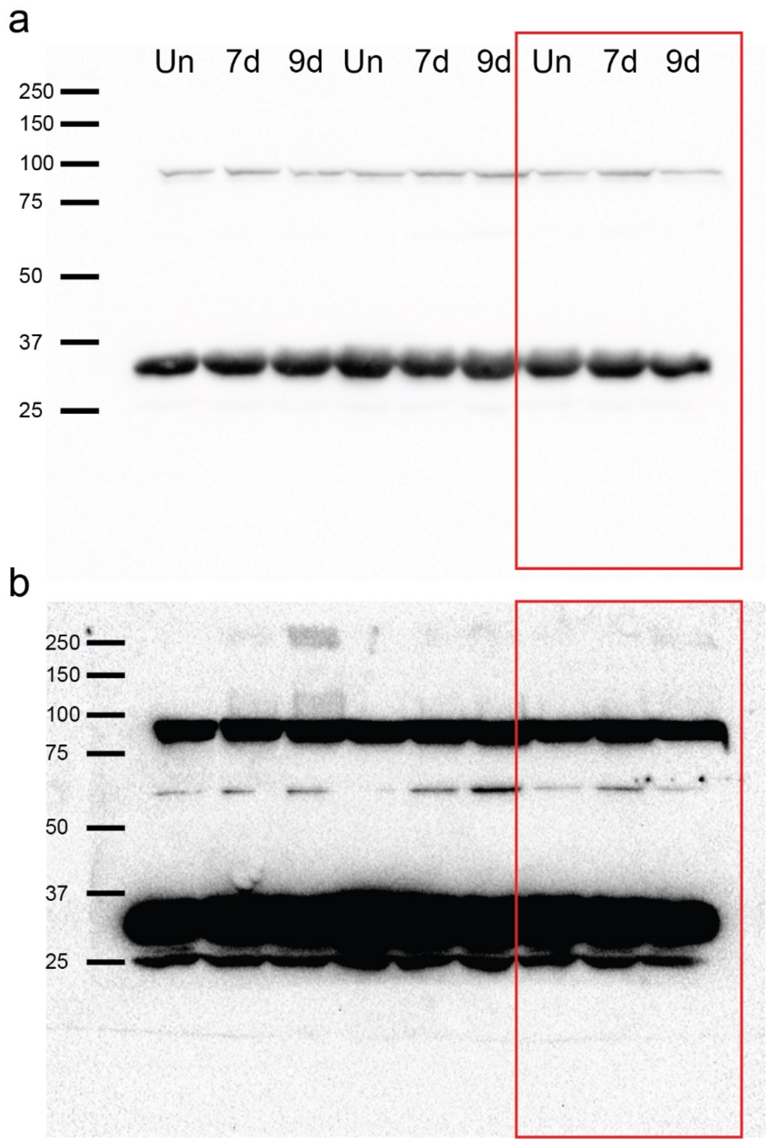

Figure S6. Uncropped images with protein size markers of Ago2/GAPDH Western blot of peroneal crush TA muscles displayed in Figure 3. (a) Original scan without changes to brightness and contrast. (b) Brightness and contrast altered to show membrane edges. Membranes were cut and probed separately for Ago2 (top) and GAPDH (bottom), then placed together for imaging. Red boxes indicate the cropped portion of the membrane displayed in figure 3.

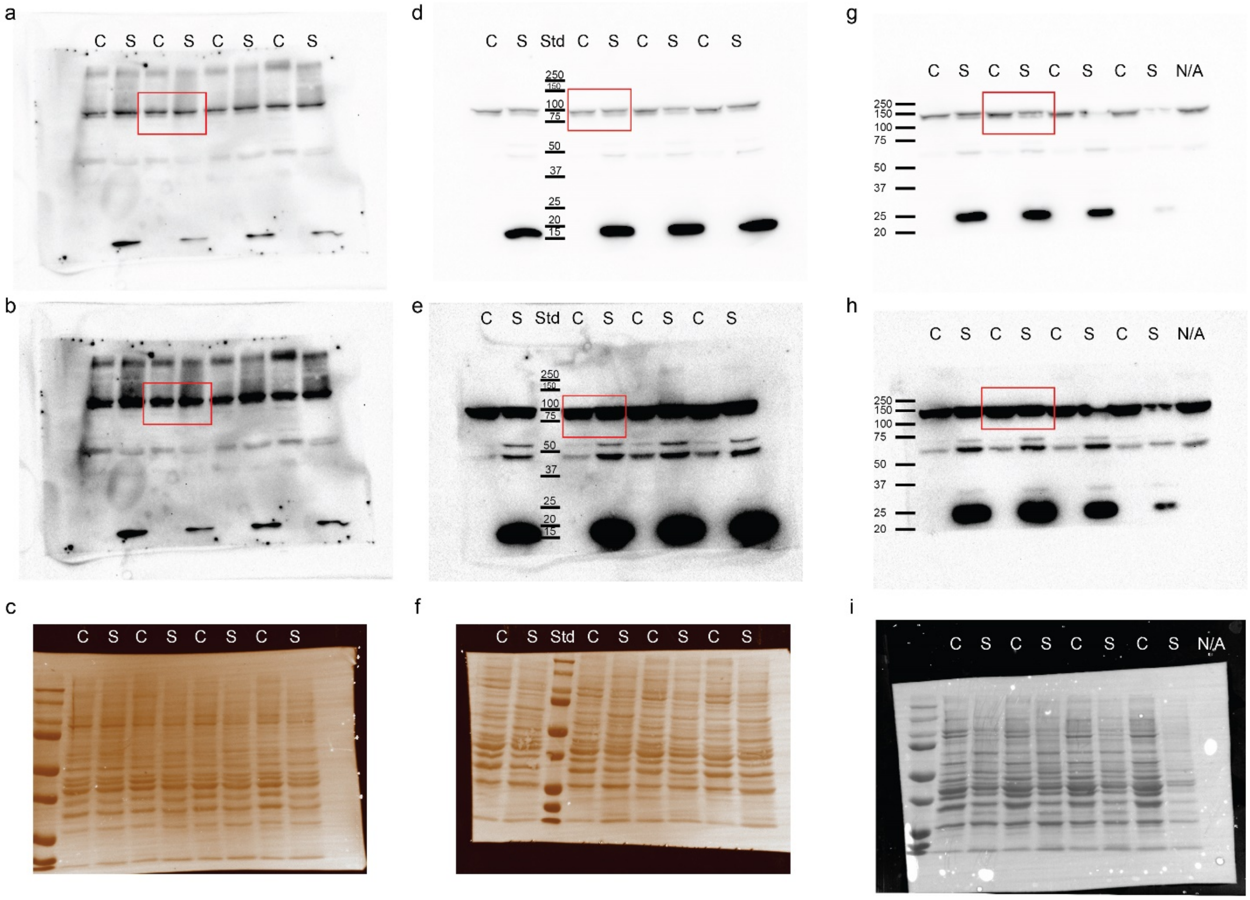

Figure S7. Uncropped images with protein size markers of Ago2 Western blot of P70 (a-c), P90 (d-f) and p110 (g-i) SOD1^{G93A} TA muscles displayed in Figure 4. (a,d,g) Original scan without changes to brightness and contrast. (b,e,h) Brightness and contrast altered to show membrane edges. (c,f,i) Representative images of Coomassie stained membranes used to determine total protein levels for densitometry normalization in Fig. 4. C = control and S = SOD1^{G93A}. Red boxes indicate the cropped portions of the membranes displayed in figure 4.

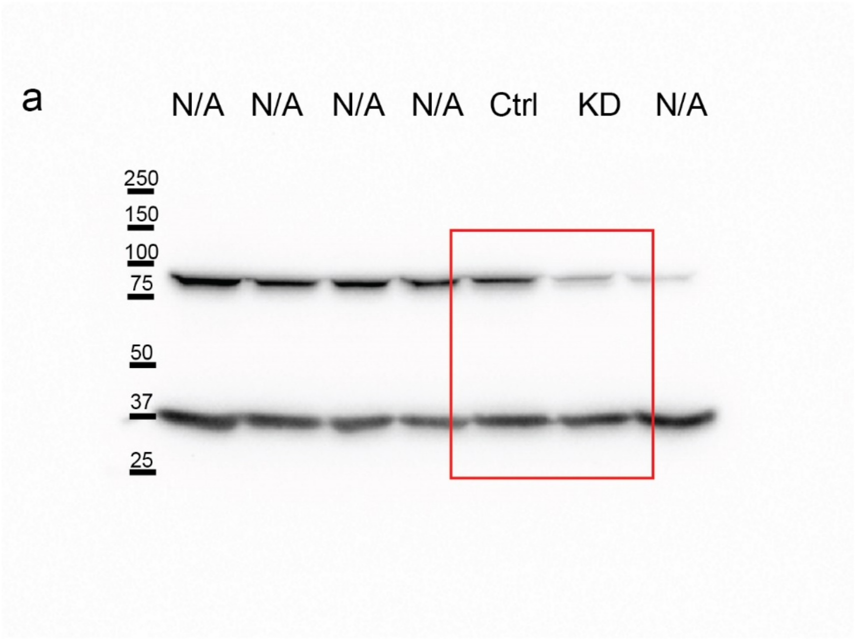

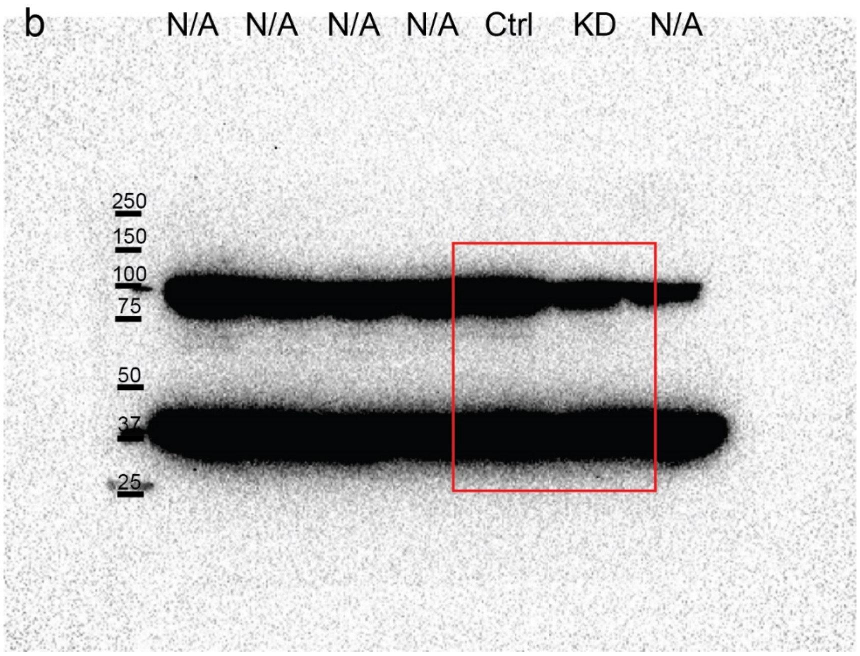

Figure S8. Uncropped images with protein size markers of Ago2/GAPDH Western blot of peroneal crush TA muscles displayed in Figure S4d. (a) Original scan without changes to brightness and contrast. (b) Brightness and contrast altered to show membrane edges. Membranes were cut and probed separately for Ago2 (top) and GAPDH (bottom), then placed together for imaging. Red boxes indicate the cropped portion of the membrane displayed in figure S4d.
